# Drug-Induced Hypertension Caused by Multikinase Inhibitors (Sorafenib, Sunitinib, Lenvatinib and Axitinib) in Renal Cell Carcinoma Treatment

**DOI:** 10.3390/ijms20194712

**Published:** 2019-09-23

**Authors:** Nanna Bæk Møller, Cecilie Budolfsen, Daniela Grimm, Marcus Krüger, Manfred Infanger, Markus Wehland, Nils E. Magnusson

**Affiliations:** 1 Department of Biomedicine, Aarhus University, Høegh-Guldbergsgade 10, 8000 Aarhus C, Denmark; nanna@ohm-it.dk (N.B.M.); cebu@clin.au.dk (C.B.); 2 Gravitational Biology and Translational Regenerative Medicine, Faculty of Medicine and Mechanical Engineering, Otto von Guericke University Magdeburg, 39120 Magdeburg, Germany; 3 Clinic for Plastic, Aesthetic and Hand Surgery, Otto von Guericke University Magdeburg, Leipziger Str. 44, 39120 Magdeburg, Germany; marcus.krueger@med.ovgu.de (M.K.); manfred.infanger@med.ovgu.de (M.I.); markus.wehland@med.ovgu.de (M.W.); 4 Medical Research Laboratory, Department of Clinical Medicine, Faculty of Health, Aarhus University, Nørrebrogade 44, 8000 Aarhus C, Denmark; nm@clin.au.dk

**Keywords:** renal cell carcinoma, sorafenib, sunitinib, lenvatinib, axitinib, multikinase inhibitor, hypertension

## Abstract

This paper reviews current treatments for renal cell carcinoma/cancer (RCC) with the multikinase inhibitors (MKIs) sorafenib, sunitinib, lenvatinib and axitinib. Furthermore, it compares these drugs regarding progression-free survival, overall survival and adverse effects (AE), with a focus on hypertension. Sorafenib and sunitinib, which are included in international clinical guidelines as first- and second-line therapy in metastatic RCC, are now being challenged by new-generation drugs like lenvatinib and axitinib. These drugs have shown significant clinical benefits for patients with RCC, but all four induce a variety of AEs. Hypertension is one of the most common AEs related to MKI treatment. Comparing sorafenib, sunitinib and lenvatinib revealed that sorafenib and sunitinib had the same efficacy, but sorafenib was safer to use. Lenvatinib showed better efficacy than sorafenib but worse safety. No trials have yet been completed that compare lenvatinib with sunitinib. Although axitinib promotes slightly higher hypertension rates compared to sunitinib, the overall discontinuation rate and cardiovascular complications are favourable. Although the mean rate of patients who develop hypertension is similar for each drug, some trials have shown large differences, which could indicate that lifestyle and/or genetic factors play an additional role.

## 1. Introduction

Hypertension is a highly prevalent and serious disease. “The 2018 ESC/ESH Guidelines for the management of arterial hypertension” [1] defined hypertension as a systolic blood pressure (SBP) ≥ 140 mmHg and a diastolic blood pressure (DBP) ≥ 90 mmHg. This condition can lead to cardiovascular diseases with fatal results [2,3].

In patients with renal cell carcinoma/cancer (RCC), new developments in targeting therapy, such as multikinase inhibitors (MKIs), have shown an improvement in progression-free survival (PFS) and overall survival (OS), and thereby an increase in the quality of life. Unfortunately, MKIs also induce numerous adverse effects (AEs), of which hypertension is a frequent occurrence [4,5,6]. Sorafenib, sunitinib, lenvatinib and axitinib are of great interest, and recent and ongoing trials are investigating these MKIs for treating RCC, with a focus on high-efficacy measures as well as optimal safety.

## 2. Methods

The literature for this review was mainly found online at PubMed [7] and ClinicalTrials [8]. The keywords searched in the literature included “Sorafenib”, “Sunitinib”, Lenvatinib”, Axitinib”, “RCC”, “renal cell carcinoma”, “renal cell cancer” and “hypertension”. The keywords were entered individually or linked together with the Boolean operator “AND”.

The following keywords were searched in PubMed: “Sorafenib AND RCC/renal cell carcinoma/renal cell cancer” returned 606/1600/1662 results; “Sorafenib AND hypertension” gave 478 results, “Sunitinib AND RCC/renal cell carcinoma/renal cell cancer” returned 912/2655/2714 results; “Sunitinib AND hypertension” provided 461 results; “Lenvatinib AND RCC/renal cell carcinoma/renal cell cancer” generated 26/72/74 results; “Lenvatinib AND hypertension” returned 78 results; “Axitinib AND RCC/renal cell carcinoma/renal cell cancer” returned 154/498/509 results; “Axitinib AND hypertension” provided 133 results (last accessed on 19 August 2019).

## 3. Renal Cell Carcinoma

### 3.1. Definition

In 2018, kidney cancer was the fourteenth most common cancer type worldwide [9,10], with an incidence rate of 406,262 new cases and 175,098 deaths per year. RCC is responsible for up to 85% of all renal neoplasms [11,12]. RCC development is linked to multiple risk factors; age and gender are strongly related to its development. The disease is more frequent in men, and the incidence rate peaks at 60–70 years of age [13]. In the early stages of RCC with a tumour size less than 30 mm, the disease is often asymptomatic and typically located by coincidence during a radiological examination. When the tumour grows larger than 30 mm, the patients may have symptoms such as fever, fatigue, weight loss, haematuria, flank mass, back pain, anaemia and high calcium levels [11].

RCC comprises a heterogenous group of tumours originating from the tubular epithelium in the kidney [9]. There are more than 10 histological RCC subtypes (Table 1). With >80% of all renal tumours, the clear cell RCC (ccRCC) represents the most common histological RCC subtype [12]. ccRCC is a highly vascular tumour characterized by malignant epithelial cells with clear cytoplasm and a compact growth pattern [14,15]. Due to its resistance to standard chemo- and radiotherapies, it has been considered as the most aggressive histological type of RCC [16]. Especially patients with metastatic ccRCC have a worse prognosis [17]. Papillary RCC (pRCC) is the second most common RCC subtype, and accounts for 10–15% of renal cortical neoplasms. pRCC tumours either show a hypovascular (tubulo)papillary architecture composed of a single layered small cell and scanty cytoplasm (type 1), or are characterized by pseudostratified large cells and an eosinophilic cytoplasm (type 2). pRCC is generally associated with a favourable outcome. Type 1 pRCC seems to have a better prognosis than type 2, but there is still no consensus regarding the standard treatment for metastatic pRCC although multiple gene mutations in pRCC could serve as a basis for targeted therapies [18]. First-line therapy for metastatic pRCC and metastatic ccRCC is generally similar. Treatment with antiangiogenic drugs such as bevacizumab, sunitinib or sorafenib could increase the progression-free survival in patients with both subtypes [19,20], although patients suffering from ccRCC showed a better response to antiangiogenic drugs [21].

**Figure 1:**
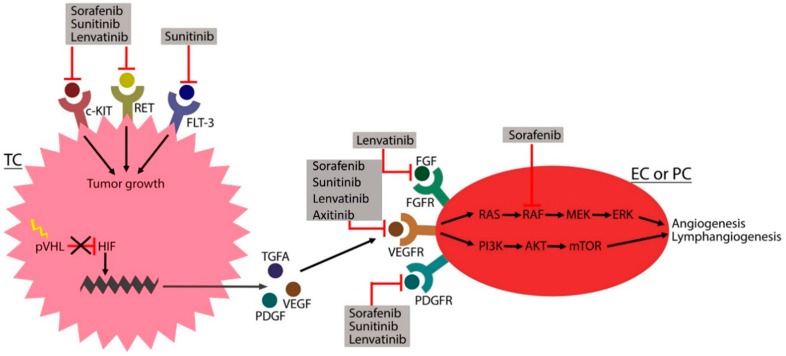
The tumorigenesis of renal cell carcinoma. VHL mutation (yellow lightning flash) reduces the amount of von Hippel Lindau protein (pVHL), and thus hypoxia-inducible factor (HIF) production is not inhibited (black cross). HIF binds the DNA (green zigzag line) and upregulates the transcription of transforming growth factor α (TGFA), vascular endothelial growth factor (VEGF) and platelet-derived growth factor (PDGF), which stimulate receptor tyrosine kinases (RTKs) on the endothelial cell (EC) or pericyte (PC) to promote angiogenesis and lymphangiogenesis. RTK activation on the tumour cell (TC) results in tumour growth. Multikinase inhibitors (MKIs), including sorafenib, sunitinib, lenvatinib and axitinib, target these RTKs to inhibit tumorigenesis. Abbreviations: FGF (fibroblast growth factor), FGFR (fibroblast growth factor receptor), VEGFR (vascular endothelial growth factor receptor), PDGFR (platelet-derived growth factor receptor), RAS (rat sarcoma), RAF (rapidly accelerated fibrosarcoma kinase), MEK (mitogen activated protein kinase), ERK (extracellular signal-regulated kinase), PI3K (phosphoinositide 3-kinase), AKT (protein kinase B), mTOR (mammalian targeted of rapamycin), c-KIT (stem cell factor receptor), RET (rearranged during transfection), FLT-3 (FMS-like tyrosine kinase 3). Arrows indicate direct relationships.

Tumourigenesis for ccRCC is highly associated with hereditary von Hippel-Lindau (VHL) disease, where the VHL tumour suppressor gene is inactivated. This change leads to lower amounts of pVHL protein and pVHL-containing protein complexes, which normally inhibit hypoxia-inducible factor (HIF). Physiologically, HIF acts as a transcription factor that upregulates the expression of vascular endothelial growth factor (VEGF), platelet-derived growth factor beta (PDGF-β) and transforming growth factor alpha [12,22,23]. These factors bind to tyrosine kinase receptors (RTKs) on the cell surface—an action that results in an intracellular activation of the Ras/Raf/mitogen activated protein kinase (MEK)/extracellular-regulated kinase (ERK) and phosphoinositide 3-kinase (PI3K)/AKT/mammalian target of rapamycin (mTOR) pathways. This activation favours the angiogenesis, survival, proliferation, differentiation and mobility of the cells. Thereby, all the essential factors for developing a tumour are fulfilled [24] (Figure 1).

### 3.2. Current Treatment Options

RCC management is based on TNM staging and pathological changes in the disease. The T describes the tumour size and the N the spread to regional lymph nodes. The M describes whether the tumour has metastasised [9,11]. Pathological gradation is ascertained by examining the cytological and histological changes found in a renal specimen. Surgical excision, either partial or radical nephrectomy, is the treatment of choice for patients with a surgically resectable RCC (Table 2). Targeted therapy and/or immunotherapy is the treatment of choice for patients with inoperable or metastatic RCC (mRCC; Table 3). RCC is resistant to chemotherapy, and thus targeted therapies are crucial [9].

### 3.3. MKIs in the Treatment of RCC

The signalling and coordination of cellular processes is initiated by protein kinases that regulate protein phosphorylation and thereby affect the location, interaction and activity of bioactive molecules. Dysregulation of the protein kinases may introduce dramatic changes in cellular processes that, in the worst case, may lead to the development of cancer or other diseases. Cancer treatment is therefore often targeted against these protein kinases, which are involved in proliferation, differentiation, cell cycle, apoptosis and angiogenesis [29]. MKIs are drugs that target multiple types of protein kinases, including vascular epithelial growth factor receptor (VEGFR), platelet-derived growth factor receptor (PDGFR), stem cell factor receptor (c-KIT) and others [4,30,31]. Signalling inhibition is initiated by drug binding to the RTK—an action that either blocks the binding site for ATP and peptide substrates or the recruitment sites of the downstream signalling substrate proteins [32]. Unfortunately, some MKIs induce AEs that negatively affect the quality of the patient’s life. One of these effects is hypertension [9].

#### 3.3.1. The Adverse Effect Hypertension

When treating RCC patients with MKIs, one of the most common AEs is hypertension (Table 4).

When using MKIs to treat a broad range of cancers, high rates of treatment-induced hypertension have been reported [37,38,39]. The definition of hypertension is an SBP ≥ 140 mmHg and a DBP ≥ 90 mmHg [1,40]. Primary/essential hypertension accounts for 95% of all cases. The overall mechanism of developing this type is unknown, but it can be influenced by obesity, amount of physical activity, arterial natriuretic peptide and the baroreflex. Secondary hypertension accounts for 5% of all hypertension cases and is caused by renal or endocrine factors (known mechanism). Hypertension is divided into categories depending on its severity [3].

Untreated hypertension is a risk factor for developing cardiovascular diseases, including stable and unstable angina, subarachnoid/intracerebral haemorrhage, myocardial infarction, heart failure, sudden cardiac death, ischaemic stroke and peripheral arterial disease, all of which show a high mortality rate [2].

Hypertension management can be achieved by either non-pharmacological or pharmacological therapy. Non-pharmacological therapy involves lifestyle changes, which focus on lower salt intake, reduced alcohol consumption, normalisation of the body mass index, no cigarette smoking and increased physical activity. If no effect is seen after six months, pharmacological therapy is warranted. For half of all patients with hypertension, non-pharmacological therapy may be able to normalise blood pressure.

Pharmacological therapy has an important role in hypertension management and treatment. Currently, five classes of antihypertensive therapy are recommended: angiotensin-converting enzyme inhibitors, angiotensin II receptor subtype 1 receptor inhibitors, β-adrenoreceptor antagonists, diuretics and calcium antagonists. If treatment produces no effect, the dose can be increased or another drug or a combination of multiple drugs can be used. Both mono- and combined therapies must be frequently controlled. After the required effect, the treatment can be reduced until discontinuation, but in most cases a lifelong therapy is needed [3].

#### 3.3.2. Induction of Hypertension

**Figure 2:**
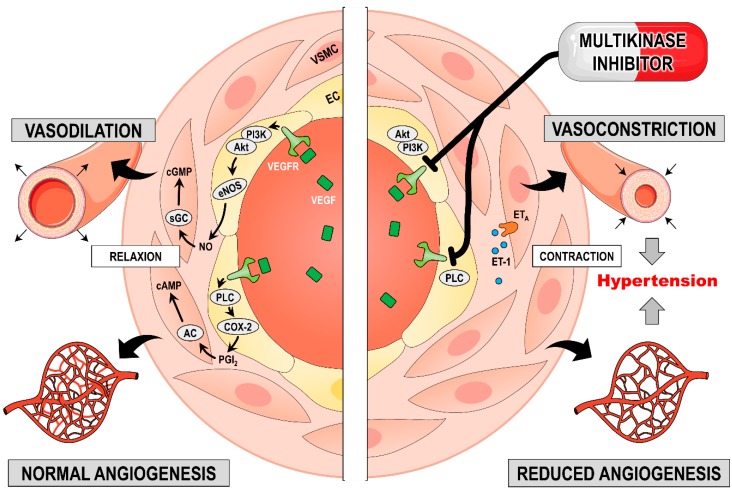
The effect of multikinase inhibitors (MKIs) on blood vessels to promote hypertension. The left panel mirrors the physiological condition and the right panel shows conditions during MKI therapy. Vascular endothelial growth factor (VEGF) binding to its receptor (VEGFR) activates phosphoinositide 3-kinase (PI3K); this binding stimulates endothelial nitric oxide synthase (eNOS) and thereby NO production. NO diffusion to vascular smooth muscle cells (VSMCs) activates guanylate cyclase (GC) to produce cyclic guanosine monophosphate (cGMP), and this action causes vessel relaxation. VEGFR also activates phospholipase C (PLC), which stimulates cyclooxygenase-2 (COX-2) to generate prostacyclin (PGI_2_). This synthesis activates adenylate cyclase (AC) in the VSMCs and leads to cyclic adenosine monophosphate (cAMP) production, which causes vessel relaxation. Blocking VEGFR inhibits NO and PGI_2_ synthesis and increases endothelin-1 (ET-1) production. This combination contracts VSMCs and promotes vessel rarefaction—a phenomenon that ultimately leads to hypertension. Additional abbreviations: EC (endothelial cell), ATP (adenosine triphosphate), GTP (guanosine triphosphate), ETA (endothelin receptor A).

The mechanism of how MKIs induce hypertension is not fully known and was recently reviewed elsewhere [39]. One explanation of how hypertension is induced is based on the production of nitric oxide (NO). Under normal conditions, binding of VEGF to VEGFR-2 on endothelial cells (ECs) results in NO production. NO diffuses to vascular smooth muscle cells (VSMCs), where it induces vasorelaxation by stimulating guanylate cyclase. VEGFR-1+2 stimulation also results in prostacyclin (PGI_2_) synthesis, which also causes vasorelaxation in VSMCs via adenylate cyclase activation [41,42]. Blocking VEGFR decreases NO and PGI_2_ synthesis and causes vasoconstriction [41]. Inhibiting VEGFRs will also increase the circulating endothelin-1 (ET-1) concentration—a change that promotes vasoconstriction. Another condition that can lead to hypertension is capillary rarefaction, a reduction in vessel density that increases vascular resistance and BP [40,41]. All these mechanisms promote vasoconstriction and will increase peripheral vascular resistance, ultimately leading to hypertension [43] (Figure 2).

#### 3.3.3. Sorafenib

**Figure 3:**
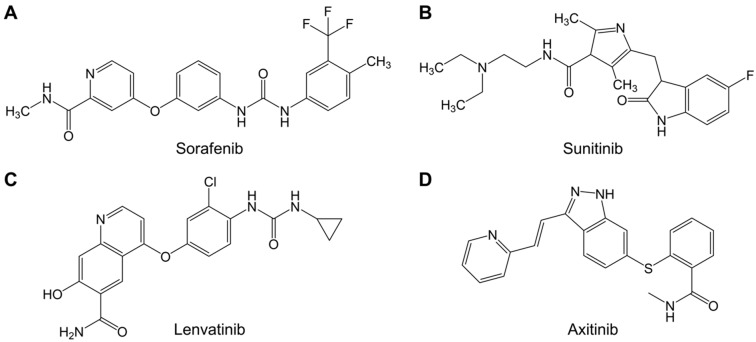
Chemical structures of (**A**) sorafenib (C_21_H_16_ClF_3_N_4_O_2_), (**B**) sunitinib (C_22_H_23_N_7_O_2_S), (**C**) lenvatinib (C_21_H_19_ClN_4_O_4_), and (**D**) axitinib (C_22_H_18_N_4_OS), modified from. The sketch was generated using ChemDraw Professional 15.0.

Sorafenib (Figure 3A) was approved by the Food and Drug Administration (FDA) in 2005 [44] and by the European Medicines Agency (EMA) in 2006 for the treatment of RCC [45].

Sorafenib inhibits RTKs, namely VEGFR-1, -2, -3, PDGFR-β, c-KIT, FMS-like tyrosine kinase-3 (FLT-3), rearranged upon transfection (RET) and the intracellular enzyme rapidly accelerated fibrosarcoma kinase (RAF) [25]. Blockage of the RTKs at the cell surface leads to inhibition of the intracellular phosphorylation cascade and the Raf/MEK/ERK and PI3K/AKT/mTOR pathways, thereby inhibiting the transcription of proteins involved in different functions. VEGFR is located on the surface of ECs, and sorafenib blocks the receptor—an action that leads to the inhibition of angiogenesis (VEGFR-1, -2) and lymphangiogenesis (VEGFR-3). Sorafenib also works as an antiangiogenic drug by inhibiting PDGFR at the surface of pericytes and smooth muscle cells. By blocking VEGFR and PDGFR, differentiation, proliferation, migration and tubular formation cannot occur. Angiogenesis is inhibited and ultimately tumourigenesis in RCC is halted. c-KIT, FLT-3 and RET are located on the tumour cell surface. Targeting these receptors will inhibit tumour growth [24,26,46]. RAF is an intracellular enzyme involved in all RTK pathways. Targeting this enzyme will thus inhibit all of the abovementioned mechanisms [24] (Figure 1). 

Sorafenib is an orally administered drug with a recommended dose of 400 mg twice a day [4]. It is absorbed from the gastrointestinal tract with 92% bioavailability [47,48]. The peak concentration of the drug (C_max_) varies from patient to patient; it occurs between 2 and 12.5 h after administration and has an elimination half-life (T_1/2_) of 20–39 h in patients with cancer. In the blood stream, >99.5% sorafenib is protein-bound, mostly to serum albumin and α-acid glycoprotein [49].

Sorafenib is transported to the liver, where it is metabolised by the enzyme CYP3A4 to an N-oxide metabolite. Sorafenib is also conjugated by UGT1A9 to sorafenib glucuronide, which can be converted back to sorafenib in the gastrointestinal tract by β-glucuronidase [47]. Excretion of the metabolites occurs via the urinary (19%) and faecal (77%) routes [50]. 

#### 3.3.4. Sunitinib

Sunitinib (Figure 3B) was FDA-approved in 2006 [44] and EMA-approved in 2006 for RCC treatment [51].

Sunitinib inhibits VEGFR-1, -2, -3, PDGFR-α, -β, c-KIT, FLT-3 and RET [25]. The mechanism of action is the same as for sorafenib; both drugs inhibit the same receptors and therefore are antiangiogenetic and antilymphangiogenetic and inhibit tumour growth [24] (Figure 1). 

Sunitinib is an orally administrated drug taken at either 37.5 or 50 mg per day for 4 weeks, followed by 2 weeks of rest [52]. After intake, it is absorbed by the gastrointestinal tract (with ≥50% bioavailability) [53]. C_max_ is reached after 6–12 h, and the T_1/2_ is 40–80 h. When transported in the blood, 65.3% binds to serum albumin and 33.7% binds to α-acid glycoprotein [54]. 

Sunitinib is transported to the liver, where it is metabolised by CYP3A4 to N-desethyl sunitinib (SU12662), which is an active metabolite. SU12662 is further metabolised by CYP3A4 to an inactive metabolite. Elimination of the metabolite occurs via the urinary (16%) and faecal (61%) routes [52].

#### 3.3.5. Lenvatinib

Lenvatinib (Figure 3C) was FDA-approved in 2015 [44] and EMA-approved in 2015 for the treatment of RCC [55].

Lenvatinib inhibits VEGFR-2, fibroblast growth factor receptor (FGFR) (1, 2, 3, 4), PDGFR-α, c-KIT and RET. Lenvatinib inhibits similar tyrosine kinases to sorafenib and sunitinib; it works as an antiangiogenic (VEGFR-2 and PDGFR-α) drug and inhibits tumour growth (c-KIT and RET). Unlike sorafenib and sunitinib, lenvatinib also inhibits FGFR located on the EC surface. FGFR activation normally stimulates migration, proliferation and tubular formation, all of which lead to angiogenesis. By inhibiting FGFR, angiogenesis is blocked (Figure 1) [24].

Lenvatinib is an orally administrated drug with a recommended dose of 24 mg per day. It is rapidly absorbed in the gastrointestinal tract with a bioavailability of 90% [56]. C_max_ is reached after 1.6 h and T_1/2_ is between 17.8 and 34.5 h [57]. Lenvatinib is primarily bound to serum albumin in the blood stream (range 97.9–98.6%) [56]. It is metabolised in both liver and kidneys and is excreted into the bile. CYP3A4 accounts for >80% of the drug elimination. The major metabolite is demethylated (M2) [58]. Excretion of the metabolites occurs via the urinary (25%) and faecal (64%) routes [57].

#### 3.3.6. Axitinib

Axitinib (Figure 3D) was FDA- and EMA-approved in 2012 as a second-line treatment for RCC [59]. Axitinib is a potent MKI that selectively inhibits VEGFR-1, -2 and -3, PDGFR-α and-β as well as c-KIT [59].

Axitinib is administered orally with a standard starting dose of 5 mg twice daily (bid). Depending on potential AEs, the dose may be increased (7 or 10 mg) later in the treatment regimen. Axitinib is absorbed rapidly, with a bioavailability of 58% and T_1/2_ between 2.5 and 6.1 h. It binds to plasma proteins (>99%), mainly serum albumin. The drug is primarily metabolised in the liver by CYP3A4; less than 1% is excreted in the urine [60].

## 4. Results

### 4.1. Clinical Trials with Sorafenib, Sunitinib, Lenvatinib and Axitinib

The currently running trials for sorafenib, sunitinib, lenvatinib and axitinib in the treatment of patients with RCC are listed in Table 5, Table 6, Table 7 and Table 8, respectively. 

Escudier et al. [33] included 903 patients previously treated for RCC who were randomly assigned to receive 400 mg sorafenib twice a day or placebo. Participants received the drug until disease progression, but some had to quit the drug due to toxicity. The median PFS was 5.5 months for patients treated with sorafenib and 2.8 months in patients receiving placebo. OS improved in sorafenib-treated patients after adjustment, with 17.8 months in the treated patients compared to 14.3 months in patients who received placebo. Even though sorafenib treatment showed notable efficacy, 87% of the patients treated with the drug developed a treatment-related AE (TRAE); hypertension developed in 17% of these patients. Only 54% of patients who received placebo developed a TRAE, and only 1% developed hypertension [33]. 

In Ravaud et al. [34], 615 participants with high-risk RCC were randomised to receive either 50 mg sunitinib per day or placebo for 4 weeks followed by 2 weeks off in one year or until disease recurrence, high grade AE or patients withdrew their consent. The median disease-free survival (DFS) was 6.8 years in patients treated with sunitinib (compared to 5.6 years in the placebo group). However, sunitinib treatment promoted a high frequency of AEs (34.3%), which led to dose reduction. Hypertension was one of the most frequent AEs, found in 44.7% of patients treated with sunitinib (of whom 7.8% presented grade 3 hypertension). In the placebo group, only 13.1% of patients developed hypertension [34]. 

In Motzer et al. [35], 153 patients with advanced RCC (ARCC) or mRCC were investigated. The patients were randomised to receive either lenvatinib (24 mg/day), everolimus (10 mg/day) or a combination of the two drugs (18 mg lenvatinib/day + 5 mg everolimus/day) in 28-day cycles. The treatment was given until disease progression, high-grade AE or patients withdrew their consent. The median PFS for lenvatinib + everolimus was 14.6 months, 7.4 months for lenvatinib and 5.5 months for everolimus only. All patients had at least one TRAE. Hypertension was seen in 41% of patients treated with the combination, 48% of patients treated with lenvatinib alone and only 10% of patients treated with everolimus [35].

In 2005, results from the first phase I axitinib trial were released. Patients with advanced solid tumours were included and the results were promising [61]. This publication was soon followed by phase II trials that investigated axitinib in the treatment of advanced RCC [36,62,63]. The study that led to FDA and EMA approval in 2012 was the randomised, open label phase III AXIS trial. A total of 723 patients with ARCC and progression on first-line therapy were included and randomised to receive either sorafenib or axitinib. The median PFS was 6.7 months in the axitinib group, compared with 4.7 months in the sorafenib group (hazard ratio 0.665; 95% confidence interval (CI) (0.544–0.812), *p* < 0.001).

Furthermore, axitinib demonstrated a favourable toxicity profile; only 14 (4%) versus 29 (8%) of patients had to discontinue treatment due to AEs. The most common AEs in the axitinib arm were diarrhoea (55%), hypertension (40%) and fatigue (39%) [64]. Grade 3 and 4 hypertension were relatively seldom, occurring in 15.3% (55/359) and 0.35% (1/359) of axitinib- and sorafenib-treated patients, respectively, and 50% of those patients continued treatment for ≥9 months. Of the 12.8% (46/359) dose interruptions, 4.5% (16/359) and 0.3% (1/359) discontinuations, respectively, were hypertension-related. Less than 1% of axitinib-treated patients suffered from hypertension-induced sequelae, and while axitinib caused hypertension more frequently than sorafenib, it rarely led to therapy discontinuation or cardiovascular complications [65].

### 4.2. Countermeasures against Drug-Induced Hypertension

Before initiating treatment with MKIs, patients should have their BP under control. Other medical conditions, including lifestyle factors known to raise BP, should be reduced [41]. If patients have confirmed hypertension, antihypertensive drugs should be offered before starting with MKIs [66].

There is no indication that antihypertensive drugs affect the anti-tumour effect of MKIs. Therefore, patients who develop hypertension (BP > 140/90) due to MKI therapy should receive standard hypertension treatments [40,41]. However, antihypertensive drugs that inhibit CYP3A4 (e.g., diltiazem or verapamil) should be avoided, because this enzyme is important for metabolising MKIs in the liver. It is very rare that patients require an MKI dose reduction or treatment discontinuation, but these measures are recommended if the BP rises to critical high grades [66]. 

### 4.3. Biomarkers in mRCC

Targeted MKI therapies have a significant role in the treatment of metastatic RCC. In the wake of personalized medicine, the need for reliable molecular biomarkers for diagnoses, prognoses and disease monitoring is rapidly increasing. One of the major challenges lies in identifying biomarkers that reach a sufficient level of clinical validation. Numerous novel single markers including circulating protein markers in blood or urine [33], micro-RNA [67,68] and tumour-derived cell-free RNA [69] have been tested with high levels of significance in RCC (Table 9). However, to our knowledge, no single marker has yet reached clinical validation, or has been shown to improve the existing prognostic models [70]. The focus has shifted towards combinations of panels of individual molecular markers with clinical markers such as neutrophil count [71,72]. Predictive biomarkers for identifying the optimal treatment on an individualized level remain a significant challenge. At present, several predictive biomarkers are under investigation, but most still require clinical validation. However, the International mRCC Database Consortium risk model was recently prospectively validated as a predictive biomarker in mRCC [73]. The model comprises six variables including neutrophil and platelet counts. It was demonstrated that this risk model could distinguish patients with mRCC into two groups that benefitted most from either immune checkpoint blockade versus sunitinib [74]. Although the AE hypertension has been associated with improved clinical outcomes on axitinib [75] and is discussed as surrogate marker for clinical efficacy of sunitinib therapy in mRCC [76], there are still no validated predictive biomarkers available.

## 5. Discussion

In recent years, targeted therapies for treating patients with RCC, including anti-VEFGR and anti-mTOR drugs, have been used as the standard of care. These drugs demonstrate prolonged PFS and OS, and thereby improve the quality of life [4].

There are a large number of clinical trials that compared the efficacy and safety of sorafenib and sunitinib in the treatment of RCC. One trial by Cai et al. [81] investigated the two drugs as first-line treatment in patients with mRCC. The PFS was 10 months (95% CI (7–13)) for sorafenib versus 11.5 months (95% CI (9–12)) for sunitinib; there was no significant difference between the two drugs. The difference in OS, 24 months (95% CI (15–31)) for sorafenib versus 23 months (95% CI (18–25)) for sunitinib, was also not significant. There were differences with regards to the number of AEs, especially the development of hypertension, which were more frequent in sunitinib-treated patients. In this study, sorafenib and sunitinib demonstrated comparable efficacy measures (PFS and OS), but sorafenib showed a more favourable profile in terms of AE development [81]. 

Comparing lenvatinib with the other drugs is challenging. For example, lenvatinib is relatively new (with regards to when it was approved), and thus only a few clinical trials have been completed that tested its effect on RCC. One trial that compared lenvatinib with sorafenib in patients with hepatocellular carcinoma was published by Kudo et al. [82]. The trial showed that the OS did not differ between the two drugs, but PFS was 7.4 months (95% CI (6.9–8.8)) for lenvatinib and only 3.7 months (95% CI (3.6–4.6)) for sorafenib (*p* < 0.001). Lenvatinib treatment resulted in a higher percentage of high-grade AEs than sorafenib. Additionally, there was a large difference in developing hypertension (42% for lenvatinib and 30% for sorafenib). One might conclude that lenvatinib has better efficacy than sorafenib but worse safety with regards to hypertension [82].

There are no published clinical trials that compared lenvatinib and sunitinib, but there is a new trial (NCT02811861) that will investigate lenvatinib + everolimus or lenvatinib + pembrolizumab versus sunitinib in patients with ARCC [8]. Overall, sorafenib and lenvatinib seem to have a similar safety and efficacy profile. 

Sorafenib was the first MKI approved for the treatment of RCC (2005). Subsequently, myriad other targeted therapies have been approved and are challenging the use of sorafenib [4]. Both axitinib and tivozanib have demonstrated a better PFS (with no differences in OS) compared to sorafenib [28,83]. Compared with temsirolimus, there is no difference in PFS, but sorafenib shows a better OS [84]. Although sorafenib is challenged based on PFS, it has a demonstrated favourable safety profile with fewer cases of hypertension compared to other MKIs [28,83] (Table 10).

Moreover, sunitinib has been compared with other MKIs. Sunitinib had better efficacy when compared to pazopanib and everolimus (anti-mTOR) [85,86]. In contrast, when sunitinib was compared to cabozantinib, sunitinib had worse efficacy, with a lower PFS and OS [87]. Regarding the safety profile, sunitinib showed a much lower percent of patients who developed hypertension compared to pazopanib and cabozantinib but not everolimus (Table 10) [85,86,87].

As mentioned earlier, lenvatinib is still a new drug on the market, so only a few clinical trials have been published testing lenvatinib with other targeted therapies. A trial of lenvatinib alone versus lenvatinib + everolimus demonstrated that the combination has promising benefits for PFS, OS and safety profile compared to lenvatinib treatment alone [35] (Table 10). 

As illustrated, one of the most common AEs associated with MKI treatment is hypertension. Different AEs have been associated with improved outcome measures [5,6]. In particular, hypertension has been suggested as a possible biomarker of treatment efficacy, and has been reviewed elsewhere [88]. Especially, sunitinib-induced hypertension and neutropenia were found to be associated with longer PFS and OS in ccRCC patients. These effects are discussed as efficacy biomarkers and as a sign of response to the MKI treatment [89]. However, data are limited by the number of studies and knowledge of the mechanism of action [88]. Table 10 compares trials and the prevalence of hypertension.

VEGFR-2 inhibition abrogates NO synthesis, VEGFR-1+2 inhibition reduces PGI_2_ synthesis and VEGFR-(1+2+3) inhibition promotes vessel rarefaction, all of which are mechanisms involved in hypertension development [41]. Both sorafenib and sunitinib target the same receptors (VEGFR-(1+2+3)), while lenvatinib only targets VEGFR-2 [4,27,41]. Thus, while sorafenib and sunitinib have the exact same targets for potentially developing hypertension, clinical trials still show a difference between the drugs [81]. Furthermore, lenvatinib caused a higher percentage of hypertension than sorafenib [82], but sorafenib targets more receptors involved in angiogenesis. Considering this finding, one might expect that sorafenib could be a stronger inducer of hypertension than lenvatinib. Therefore, it is very important to note that other conditions like lifestyle or genetic factors may also play a role in this phenomenon.

## 6. Conclusions and Outlook

The evolution of targeted therapies has changed PFS and OS for patients suffering from RCC and thereby increased their quality of life. Of these targeted therapies, MKIs (especially those that target VEGFRs) inhibit angiogenesis. Sorafenib, sunitinib, lenvatinib and axitinib have all demonstrated improvements in RCC treatment. Sorafenib and sunitinib are currently being challenged by newer targeted therapies like lenvatinib, axitinib and mTOR inhibitors, among others.

A major AE when administering MKIs is hypertension. Hypertension development is linked to the VEGFR targeting, which inhibits NO and PGI_2_ production, induces ET-1, and promotes vasocontraction and vessel rarefaction [31]. However, the complete mechanism is not yet fully understood. There is no report about complications when patients are treated with antihypertensive drugs together with an MKI. Only in a few cases with high-grade hypertension was it necessary to decrease the dose or totally stop MKI therapy. The number of reported AEs is quite variable among clinical trials, suggesting that lifestyle and other predisposing factors may also influence AE (especially hypertension) development and should be studied in detail in the future.

Currently, numerous trials that are investigating MKI efficacy and safety for RCC treatment are recruiting participants. The combination of anti-VEGFR and anti-mTOR is a promising treatment (e.g., lenvatinib + everolimus), and perhaps in the future more combined targeted therapy regimens will be used in cancer. The main goal is to develop a drug with great efficacy and safety with the least amount of toxicity.

## Figures and Tables

**Table 1:** Classification of histological renal cell carcinoma (RCC) subtypes (modified from Randall [12]).

RCC Subtype	Percent of RCC Cases
Clear cell	>80%
Papillary	10–15%
Chromophobe	5%
Collecting duct	<1%
Medullary	Rare
Mucinous	Rare
Xp11	Rare

**Table 2:** Surgical treatment for renal cell carcinoma [9].

	Tumour Stage	Surgery
Partial nephrectomy	T1	Complete removal of the primary tumour, leaving the largest amount of healthy renal tissue
Radical nephrectomy	T1 and T2 Tumour ≤ 5 cm in the inferior pole	Removal of the renal, perirenal fat tissue, adrenal gland and regional lymph nodes

**Table 3:** Medical management of renal cell carcinoma [4,24,25,26,27,28].

Drug	Pathway Interaction	Treatment Target	Mechanism	Administration
Targeted therapies				
Sorafenib	Multikinase inhibition	VEGFR (1, 2, 3), PDGFR (α + β), Raf, C-KIT, RET	Anti-angiogenetic, anti-lymphangiogenic, inhibition of tumour growth	p. o.
Sunitinib	Multikinase inhibition	VEGFR (1, 2, 3), PDGFR (α + β), c-KIT, FLT-3, RET	Anti-angiogenetic, anti-lymphangiogenic, inhibition of tumour growth	p. o.
Pazopanib	Multikinase inhibition	VEGFR (1, 2, 3), PDGFR (α + β), RET, c-KIT	Anti-angiogenetic, anti-lymphangiogenic, inhibition of tumour growth	p. o.
Axitinib	Multikinase inhibition	VEGFR (1, 2, 3), c-KIT, PDGFR-β	Anti-angiogenetic, anti-lymphangiogenic, inhibition of tumour growth	p. o.
Lenvatinib	Multikinase inhibition	VEGFR-2, FGFR (1, 2, 3, 4), PDGFR-α, c-KIT, RET	Anti-angiogenetic, inhibition of tumour growth	p. o.
Tivozanib	Multikinase inhibitor	VEGFR (1, 2, 3), PDGFR-β, c-KIT	Anti-angiogenetic, anti-lymphangiogenic, inhibition of tumour growth	p. o.
Cabozantinib	Multikinase inhibition	MET, VEGFR-2, RET	Anti-angiogenetic, inhibition of cell migration and invasion	p. o.
Everolimus	mTOR inhibition	mTOR, HIF(1–2), VEGF	Cellular metabolism, cell growth, apoptosis and angiogenesis regulation	p. o.
Temsirolimus	mTOR inhibition	mTOR, HIF(1–2), VEGF	Cellular metabolism, cell growth, apoptosis and angiogenesis regulation	I.V.
Bevacizumab	Anti-VEGF monoclonal antibody	VEGF	Anti-angiogenetic	I.V.
Immunotherapy				
Interferon-α	Immune system activation	Leucocytes	Immunologic, antiproliferation, antiviral and antiangiogenic	I.V.
High-dose IL-2	Immune system activation	Leucocytes	Activation of the immune system leading to tumour regression	I.V.
Nivolumab	Programmed death 1 (PD-1)-antibody	PD-1 at T-lymphocytes	Blocking the PD-1/PD-L1 resulting in cellular immune response inhibition and restoration of antitumour immunity	I.V.

Abbreviations: p.o. (per os), I.V. (intravascular), VEGFR (vascular endothelial growth factor receptor), PDGFR (platelet-derived growth factor receptor), c-KIT (stem cell factor receptor), RET (rearranged during transfection), FLT-3 (FLT-like tyrosine kinase-3), FGFR (fibroblast growth factor receptor), mTOR (mammalian target of rapamycin), HIF (hypoxia-inducible factor).

**Table 4:** Adverse effects of the multikinase inhibitors sunitinib, sorafenib, lenvatinib and axitinib [33,34,35,36].

Drug	Adverse Effects (10 Most Common)
Sorafenib	Diarrhoea, rash, hand–foot syndrome, alopecia, fatigue, nausea, hypertension, pruritus, dry skin, vomiting
Sunitinib	Diarrhoea, hand–foot syndrome, hypertension, fatigue, dysgeusia, mucositis, dyspepsia, stomatitis, neutropenia
Lenvatinib	Diarrhoea, nausea, decreased appetite, hypertension, weight loss, fatigue, vomiting, hypothyroidism, abdominal pain, stomatitis
Axitinib	Diarrhoea, hypertension, fatigue, decreased appetite, nausea, dysphoria, palmar–plantar erythrodysesthesia, weight loss, vomiting, asthenia

**Table 5:** Most recent sorafenib clinical trials for treating renal cell carcinoma [8].

Title and ClinicalTrials.Gov NCT Number	Design and Study Size	Objective	Status and Conclusion
“A multicenter uncontrolled study of sorafenib in patients with unresectable and/or metastatic renal cell carcinoma” NCT00586105	Interventional, non-randomised, open-label, multicentre, phase III study; 39 participants	This trial investigated the efficacy, safety, tolerability and pharmacokinetic profile of sorafenib in patients with an unresectable and/or mRCC.	Completed The trial showed a PFS of 5.5 months (95% CI [4.1–7.4]) and an OS of 7.8 months (95% CI [0.9–13.4]). Hypertension was reported in 17.95% of the patients.
“A phase III randomized study of BAY43-9006 in patients with unresectable and/or metastatic renal cell cancer” NCT00073307	Interventional, randomised, parallel assignment, phase III; 903 participants	This study investigated the efficacy, safety and pharmacokinetics of patients with unresectable and/or mRCC treated with sorafenib.	Completed Sorafenib treatment in patients with mRCC showed significant improvement compared to the placebo group. Hypertension developed in 16.41% of the patients who received sorafenib.
“Sorafenib dose escalation in treatment-naïve patients with metastatic renal cell carcinoma: a non-randomized, open-label, Phase 2b study” NCT00618982	International, non-randomised, open label, uncontrolled, multicentre phase IIb study; 83 participants	This study investigated the efficacy and safety of sorafenib in patients with mRCC who had no prior systemic treatment.	Completed Patients treated with sorafenib showed clinical benefits. Hypertension was found in 48.2% of the patients.

Abbreviations: mRCC (metastatic renal cell carcinoma), PFS (progression-free survival), OS (overall survival), CI (confidence interval).

**Table 6:** Currently active clinical trials on the use of sunitinib for renal cell carcinoma therapy [8].

Title and ClinicalTrials.Gov NCT Number	Design and Study Size	Objective	Status
“A study of abemaciclib in combination with sunitinib in metastatic renal cell carcinoma” NCT03905889	Interventional, non-randomised, open label phase Ib study; 22 participants	To investigate the combination of abemaciclib with sunitinib. Primary outcome measures: Maximal tolerated dose and toxicity and pharmacokinetic assessment.	Recruiting
“A study of NKTR-214 in combination with nivolumab compared with the investigator’s choice of a tyrosine kinase inhibitor (TKI) therapy (either sunitinib or cabozantinib monotherapy) for advanced metastatic renal cell carcinoma” (RCC) NCT03729245	Interventional, randomised, open label phase III trial; 600 participants	To investigate NKTR-214 in combination with nivolumab compared to the investigator’s choice of TKI (including sunitinib). Primary outcome measures: ORR and OS.	Recruiting
“A study of nivolumab combined with cabozantinib compared to sunitinib in previously untreated advanced or metastatic renal cell carcinoma (CheckMate 9ER)” NCT03141177	Interventional, randomised, open label phase III trial; 638 participants	This study investigates the efficacy and safety of nivolumab combined with cabozantinib compared to sunitinib. Primary outcome measure: PFS. Secondary outcome measures: OS, ORR and AEs.	Active, not recruiting.
“Real-world clinical patterns of care and outcomes among mRCC patients receiving sunitinib as first line therapy. (OPTIMISE)” NCT03140176	Observational, prospective study; 140 participants	This study aims to investigate efficacy, adverse events and health-related quality of life in patients receiving sunitinib. Primary outcome measures: PFS and time to failure (TTF).	Recruiting
“Biomarker study of patients with metastatic ccRCC undergoing sequential therapy with 1st line sunitinib and 2nd line axitinib (SuAx)” NCT03592199	Interventional, open label phase II study; 30 participants	To investigate potential prognostic and/or predictive biomarkers. Primary outcome measure: RR.	Recruiting
“Study to evaluate efficacy and safety of sunitinib in renal cell carcinoma progressed to 1L immunotherapy treatment. (INMUNOSUN)” NCT03066427	Interventional, open label phase II study; 23 participants	To investigate the activity of sunitinib after treatment with new immunotherapy regimens that are currently developed in phase III trials. Primary outcome measure: ORR. Secondary outcome measures: time to progression (TTP), duration of response, OR, etc.	Recruiting
“Cabozantinib or sunitinib malate in treating participants with metastatic variant histology renal cell carcinoma” NCT03541902	Interventional, randomised, open label phase II trial; 84 participants	To compare the safety and efficacy of cabozantinib and sunitinib. Primary outcome measure: PFS. Secondary outcome measures: ORR, OS, AE rate.	Recruiting
“Alternative schedule sunitinib in metastatic renal cell carcinoma: cardiopulmonary exercise testing (ASSET)” NCT03109015	Interventional, randomised, open label phase II trial; 30 participants	To compare sunitinib administration of a 2/1 schedule (2 weeks of treatment followed by 1 week without) to a 4/2 schedule (4 weeks of treatment followed by 2 weeks without) on cardiopulmonary function. Primary outcome measure: Change in VO2 peak from baseline to week 12.	Recruiting
“Role of PRoactivE Coaching on PAtient REported outcome in advanced or metastatic RCC treated with sunitinib (PREPARE)” NCT03013946	Interventional, randomised, open label phase III study; 430 participants	To evaluate the effect of a 24-week concomitant coaching program. Primary outcome measure: Quality of life assessment.	Recruiting
“Study of patients with metastatic and/or advanced renal cell carcinoma, treated with sunitinib/axitinib.” NCT04033991	Observational, retrospective cohort study; 841 participants	To investigate patients treated with first-line sunitinib and second-line axitinib. Primary outcome measures: PFS (first-line treatment with sunitinib) and PFS (second-line treatment with axitinib).	Not yet recruiting
“Impact of sunitinib bioavailability on toxicity and treatment efficacy in patients treated for metastatic renal cancer (BIOSUNTOX)” NCT03846128	Observational, prospective cohort study; 64 participants	To measure the plasma concentration of sunitinib and its active metabolite desethyl-sunitinib (DES) and evaluate the safety and efficacy at the end of each cycle. Primary outcome measures: Hazard ratio for severe toxicity (grade 3–4 clinical and/or biological) according to the plasma sunitinib concentration.	Not yet recruiting
“Registry of complete responses to sunitinib in Spanish patients with metastatic renal cell carcinoma (ATILA)” NCT03916458	Observational, retrospective study; 90 participants	To investigate patients treated with first-line sunitinib who obtained complete response. Primary outcome measures: Percentage of patients with a good, intermediate and poor prognosis who achieved complete response in the investigator′s opinion or at least two consecutive CT scans.	Not yet recruiting
“A biomarker driven trial with nivolumab and ipilimumab or VEGFR tKi in naïve metastatic kidney cancer (BIONIKK)” NCT02960906	Interventional, randomised, open label phase II study; 150 participants	To compare nivolumab monotherapy, nivolumab combined with ipilimumab, or TKI: sunitinib or pazopanib. Primary outcome measures: ORR evaluation according to molecular groups (ccRCC1 to 4) and assigned treatment.	Recruiting
“Quality of life assessment in daily clinical oncology practice for patients with advanced renal cell carcinoma (QUANARIE)” NCT03062410	Interventional, open label study; 56 participants	To investigate and evaluate the use of HRQoL assessment in daily clinical practice. Primary outcome measure: Rate of completed questionnaires at 12 months. Secondary outcome measures: Exhaustiveness, acceptability, effectiveness and physician satisfaction.	Recruiting
“Savolitinib vs. sunitinib in MET-driven PRCC” NCT03091192	Interventional, randomised, open label phase III study; 60 participants	To compare savolitinib to sunitinib in Mesenchymal Epithelial Transition (MET)-driven papillary renal cell carcinoma. Primary outcome measure: PFS. Secondary outcome measures: OS, ORR, DoR, etc.	Active, not recruiting
“Evaluation of a promising new combination of protein kinase inhibitors on organotypic cultures of human renal tumours (COMBOREIN)” NCT03571438	Interventional, non-randomised, open label study; 100 participants	To compare the treatment of a cell culture with a combination of CK2 and ATM inhibitors serine/threonine kinase combination with sunitinib, pazopanib and temsirolimus. Primary outcome measures: Death cell rate on organotypic cultures of human renal tumours.	Recruiting

Abbreviations: TKI (tyrosine kinase inhibitor), ORR (objective response rate), OS (overall survival), PFS (progression-free survival), AE (adverse event), TFF (time to failure), RR (response rate), CT (computed tomography), ccRCC (clear cell renal carcinoma), HRQoL (health-related quality of life), DoR (duration of response), CCK (cholecystokinin), ATM (ataxia-telangiectasia mutated).

**Table 7:** Currently active clinical trials on the use of lenvatinib for treating renal cell carcinoma [8].

Title and ClinicalTrials.Gov Number	Design and Study Size	Objective	Status
“Trial to assess safety and efficacy of lenvatinib in combination with everolimus in participants with renal cell carcinoma” NCT03173560	Interventional, randomised, open label phase II study; 338 participants	To compare and evaluate the efficacy and safety of two different treatment regimens with lenvatinib in combination with everolimus. Primary outcome measures: ORR and percentage of participants with intolerable Grade 2 or any ≥Grade 3 TEAEs.	Recruiting
“Lenvatinib and everolimus in renal cell carcinoma (RCC)” NCT03324373	Interventional, open label phase I study; 15 participants	To investigate the use of lenvatinib in combination with everolimus in orally advanced and metastatic renal cell carcinoma, prior to cytoreductive nephrectomy.	Recruiting
“A phase 2 trial to evaluate efficacy and safety of lenvatinib in combination with everolimus in subjects with unresectable advanced or metastatic non clear cell renal cell carcinoma (nccRCC) who have not received any chemotherapy for advanced disease” NCT02915783	Interventional, open label phase II study; 31 participants	To investigate lenvatinib in combination with everolimus in patients with unresectable advanced or metastatic nccRCC and who have not received prior chemotherapy for advanced disease. Primary outcome measure: ORR. Secondary outcome measures: PFS and OS.	Active, not recruiting
“Phase 1b trial of lenvatinib plus pembrolizumab in subjects with selected solid tumours” NCT03006887	Interventional, open label phase I study; 6 participants	To investigate the safety of lenvatinib in combination with pembrolizumab in patients with selected solid tumours (primarily clear cell renal cell carcinoma). Primary outcome measures: AE and DLT.	Active, not recruiting

Abbreviations: ORR (objective response rate), TEAE (treatment-emergent adverse event), nccRCC (non-clear cell renal cell carcinoma), PFS (progression-free survival), OS (overall survival), AE (adverse event), DLT (dose-limiting toxicity).

**Table 8:** Currently active clinical trials for the use of axitinib in renal cell carcinoma therapy [49].

Title and ClinicalTrials.Gov NCT Number	Design and Study Size	Objective	Status
“Study of nivolumab and axitinib in patients with advanced renal cell carcinoma” NCT03172754	Interventional, non-randomised, open label phase I/II study; 98 participants	To investigate axitinib in combination with nivolumab. Primary outcome measures: Incidence of treatment-related adverse events, ORR.	Recruiting
“Study of axitinib for reducing extent of venous tumour thrombus in renal cancer with venous invasion (NAXIVA)” NCT03494816	Interventional, open label phase II study; 20 participants	To investigate axitinib in patients with metastatic and non-metastatic renal cell carcinoma and venous invasion. Primary outcome measure: Improvement in Mayo classification.	Recruiting
“Biomarker study of pts with metastatic ccRCC undergoing sequential therapy with 1^st^ line sunitinib and 2nd line axitinib (SuAx)” NCT03592199	Interventional, open label phase II study; 30 participants	To investigate potential prognostic and/or predictive biomarkers. Primary outcome measure: RR.	Recruiting
“Axitinib with or without anti-ox40 antibody pf-04518600 in treating patients with metastatic kidney cancer” NCT03092856	Interventional, randomised, double-blind phase II study; 104 participants	To investigate axitinib in combination with anti-OX40 antibody PF-04518600 compared to axitinib and placebo. Primary outcome measure: PFS. Secondary outcome measures: Incidence of unacceptable toxicity and ORR.	Recruiting
“Axitinib and nivolumab in treating patients with unresectable or metastatic TFE/translocation renal cell carcinoma” NCT03595124	Interventional, randomised, open label phase II study; 87 participants	To investigate the efficacy of axitinib in combination with nivolumab in treating unresectable or metastatic TFE/translocation renal cell carcinoma. Primary outcome measure: PFS.	Recruiting
“Prior axitinib as a determinant of outcome of renal surgery (PADRES)” NCT03438708	Interventional, open label phase II study; 50 participants	To evaluate axitinib in patients with strong PN indication (but it cannot be done due to anatomic considerations or concerns of residual renal function). Primary outcome measures: Reduction in tumour diameter, ORR, effect on tumour morphometry and feasibility of PN.	Recruiting
“Neoadjuvant axitinib and avelumab for patients with localized clear-cell RCC” NCT03341845	Interventional, open label phase II study; 40 participants	To investigate axitinib in combination with avelumab in patients with intermediate to high-risk non-metastatic RCC. Primary outcome measure: Number of patients with partial remission.	Recruiting
“Study of patients with metastatic and/or advanced renal cell carcinoma, treated with sunitinib/axitinib” NCT04033991	Observational, retrospective cohort study; 841 participants	To investigate patients treated with first-line sunitinib and second-line axitinib. Primary outcome measures: PFS (first-line treatment with sunitinib) and PFS (second-line treatment with axitinib).	Not yet recruiting
“Tolerability and pharmacokinetics of toripalimab in combination with axitinib in patients with kidney cancer and melanoma” NCT03086174	Interventional, open label phase Ib study; 24 participants	To investigate dose-escalation, tolerability and pharmacokinetics study evaluating anti-PD-1 mAb for injection in combination with axitinib in patients with advanced kidney cancer and melanoma who have failed in routine systemic treatment.	Not yet recruiting
“Post marketing surveillance study to observe safety and efficacy of Inlyta in South Korea” NCT02156895	Observational, prospective case-only study; 100 participants	To evaluate the efficacy and safety of axitinib in advanced renal cell carcinoma. Primary outcome measure: Adverse event incidence.	Recruiting
“A study of Anti-PD-1 combinations of D-CIK immunotherapy and axitinib in advanced renal carcinoma” NCT03736330	Interventional, open label phase II study; 24 participants	To investigate the safety and efficacy of immunotherapy (anti-PD-1 (pembrolizumab) activated D-CIK) in combination with axitinib. Primary outcome measure: ORR. Secondary outcome measures: PFS, OS, DoR, etc.	Recruiting

Abbreviations: ORR (objective response rate), RR (response rate), PFS (progression-free survival), PN (partial nephrectomy), DoR (duration of response).

**Table 9:** Extract of recent updates for biomarkers in RCC.

Study	References
International mRCC Database Consortium (IMDC) risk model	Heng et al. 2013 [77], Dudani et al. 2019 [73], Graham et al. 2018 [78],
C-reactive protein and the neutrophil-to-lymphocyte ratio are prognostic biomarkers in mRCC	Suzuki et al. 2019 [79]
MiR-376b-3p Is Associated with Long-term Response to Sunitinib in Metastatic Renal Cell Carcinoma Patients	Kovacova et al. 2019 [80]
34betaE12 Immuno-Expression in Clear Cell Papillary RCC	Martignoni et al. 2017 [67]
MiR-144-3p Plasma Diagnostic Biomarker for ccRCC.	Lou et al. 2017 [68]
Circulating Biomarkers to Guide Antiangiogenic and Immune Therapies	Zhang et al. 2016 [69]
Tissue-Based Biomarker Signature in ccRCC	Haddad et al. 2017 [71]
Identification of ccRCC Using a Three-Gene Promoter Methylation Panel	Pires-Luis et al. 2017 [72]

Abbreviations: mRCC (metastatic renal cell carcinoma), ccRCC (clear cell renal cell carcinoma), 34betaE12 (antibody with specificity for cytokeratins 1, 5, 10 and 14), MiR (micro RNA).

**Table 10:** Overview of trials that investigated axitinib, sorafenib, sunitinib and lenvatinib with other targeted therapies: special focus on hypertension.

Trial	Drugs	PFS (Months (95% CI))	OS (Months (95% CI))	Hypertension (%)
Motzer et al. [83]	sorafenib vs. axitinib	5.7 (4.7–6.5) vs. 8.3 (6.7–9.2) *p* < 0.0001	19.2 (17.5–22.3) vs. 20.1 (16.7–23.4) *p* = 0.3744	30 vs. 42
Hutson et al. [84]	sorafenib vs. temsirolimus	3.9 (2.8–4.2 vs. 4.3 (4–5.4) *p* = 0.19	16.6 (13.6–18.7) vs. 12.3 (10.1–14.8) *p* = 0.01	Not reported
Motzer et al. [28]	sorafenib vs. tivozanib	9.1 (7.3–9.5) vs. 11.9 (9.3–14.7) *p* = 0.042	29.3 vs. 28.8 *p* = 0.105	34 vs. 44
Motzer et al. [85]	sunitinib vs. pazopanib	9.5 (9.3–11.1) vs. 8.4 (8.3–10.9)	29.3 (25.3–32.5) vs. 28.4 (26.2–35.6) *p* = 0.28	40.5 vs. 46.21
Choueiri et al. [87]	sunitinib vs. cabozantinib	5.6 (3.4–8.1) vs. 8.2 (6.2–8.8) *p* = 0.012	21.8 (16.3–27.0) vs. 30.3 (14.6–35)	68.1 vs. 80.8
Armstrong et al. [86]	sunitinib vs. everolimus	8.3 (5.8–11.4) vs. 5.6 (5.5–6) *p* = 0.16	31.5 (14.8–NR) vs. 13.2 (9.7–37.9) *p* = 0.60	46 vs. 2
Motzer et al. [35]	lenvatinib vs. lenvatinib + everolimus	7.4 (5.6–10.2) vs. 14.6 (5.9–20.1) *p* = 0.12	18.4 (13.3–NR) vs. 25.5 (20.8–25.5) *p* = 0.32	48 vs. 41

Abbreviations: PFS (progression-free survival), OS (overall survival), CI (confidence interval), NR (not reported).

## Abbreviations

AC	adenylate cyclase
AE(s)	adverse effect(s)
AKT	protein kinase B
ARCC	advanced renal cell carcinoma
ATP	adenosine triphosphate
cAMP	cyclic adenosine monophosphate
ccRCC	clear cell renal cell carcinoma
cGMP	cyclic guanosine monophosphate
CI	confidence interval
C-KIT	stem cell factor receptor
C_max_	peak concentration of a drug
COX-2	cyclooxygenae-2)
CYP3A4	cytochrome P450 3A4
DBP	diastolic blood pressure
DSF	disease-free survival
EC	endothelial cell
EMA	European Medicines Agency
eNOS	endothelial nitric oxide synthase
ERK	extracellular signal-regulated kinase
ET-1	endothelin-1
ETA	endothelin receptor A
FDA	Food and Drug Administration
FGF	fibroblast growth factor
FGFR	fibroblast growth factor receptor
FLT-3	FMS-like tyrosine kinase 3
GC	guanylyl cyclase
GTP	guanosine triphosphate
HIF	hypoxia-inducible factor
I.V.	intravenous
MEK	Mitogen-activated protein kinase
MET	hepatocyte growth factor receptor
MKI(s)	multikinase inhibitor(s)
mRCC	metastatic renal cell carcinoma/cancer
mTOR	mammalian target of rapamycin
NO	nitric oxide
OS	overall survival
PC	pericyte
PD-1	programmed death-1
PDGF	platelet-derived growth factor
PDGFR	platelet-derived growth factor receptor
PFS	progression-free survival
PGI_2_	prostacyclin
PI3K	phosphoinositide 3-kinase
PLC	phospholipase C
pVHL	von Hippel-Lindau protein
RCC	renal cell carcinoma
RAF	rapidly accelerated fibrosarcoma kinase
RAS	rat sarcoma
RET	rearranged during transfection
RTK	tyrosine kinase receptor
SBP	systolic blood pressure
TC	tumour cell
TGFA	transforming growth factor alpha
TRAE	treatment-related adverse effect
TTP	time to progression
T_1/2_	elimination half-life
UGT1A9	uridine diphosphate glucuronosyltransferase 1A9
VEGF	vascular endothelial growth factor
VEGFR	vascular endothelial growth factor receptor
VHL	von Hippel-Lindau
VSMC	vascular smooth muscle cell
